# Solitary fibrous tumor of the pleura with marked cystic degeneration: a case report

**DOI:** 10.1186/s40792-020-00931-9

**Published:** 2020-07-07

**Authors:** Takuya Watanabe, Masayuki Tanahashi, Eriko Suzuki, Naoko Yoshii, Kensuke Chiba, Hiroyuki Tsuchida, Shogo Yobita, Suiha Uchiyama, Kensuke Iguchi, Hiroshi Ogawa, Hiroshi Niwa

**Affiliations:** 1 Division of Thoracic Surgery, Respiratory Disease Center, Seirei Mikatahara General Hospital, 3453, Mikatahara-cho, Kita-ku Hamamatsu, Shizuoka, Japan; 2 Division of Pathology, Seirei Mikatahara General Hospital, 3453, Mikatahara-cho, Kita-ku Hamamatsu, Shizuoka, Japan

**Keywords:** Solitary fibrous tumor, Cyst, Cystic degeneration, Pleura

## Abstract

**Background:**

Solitary fibrous tumor of the pleura (SFTP) is a mesenchymal tumor, and computed tomography typically shows SFTPs as well-defined lobulated masses. We herein report a case of SFTP with cystic degeneration of the entire tumor.

**Case presentation:**

The patient was a 67-year-old Japanese man who was referred to our hospital for an abnormal shadow on a chest X-ray. Computed tomography showed a 9-cm cystic tumor in the lower left lobe, with small nodules aggregated in the cyst. Pulmonary aspergillosis was suspected, and left basal segmentectomy was performed. A pedunculated cystic tumor was connected to the pleura with a stalk, and white polypoidal masses were found within the cystic tumor. Microscopy revealed uniform fibroblastic spindle cell proliferation and marked cystic degeneration, the cyst walls were formed of the same tumor cells. Immunohistochemical staining revealed that the tumor cells were positive for CD34, CD99, and BCL2. Based on these findings, the tumor was diagnosed as SFTP with cystic degeneration.

**Conclusion:**

We experienced an extremely rare case of atypical SFTP with cystic degeneration.

## Background

Solitary fibrous tumors of the pleura (SFTPs) are relatively rare, accounting for < 5% of all pleural tumors [1]. Chest radiography of patients with SFTP typically demonstrates a well-defined, solitary nodule or mass, which may appear at the lung periphery and which typically abuts the pleural surface [2]. Chest computed tomography (CT) is the key examination for diagnostic imaging and surgical planning. In most cases of SFTP, CT demonstrates a homogeneous well-defined, non-invasive, lobular, soft-tissue mass, adjacent to the chest wall, or within a fissure, showing an obtuse angle with the pleural surface [3]. In contrast to these typical findings, we report an extremely rare case of SFTP with cystic degeneration.

## Case presentation

**Fig. 1:**
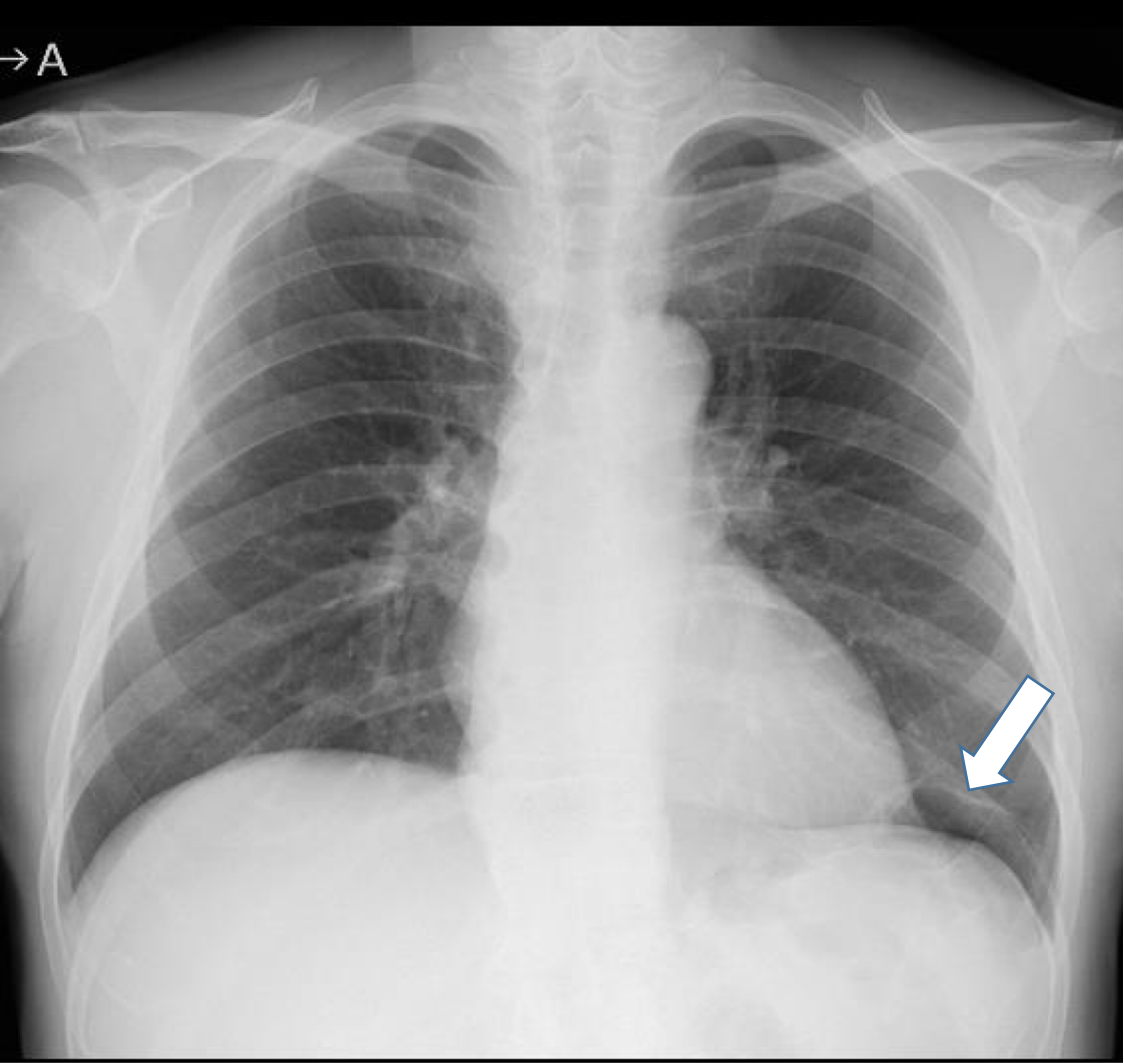
A chest radiograph showed a linear shadow in the left lower lung field (arrow)

**Fig. 2:**
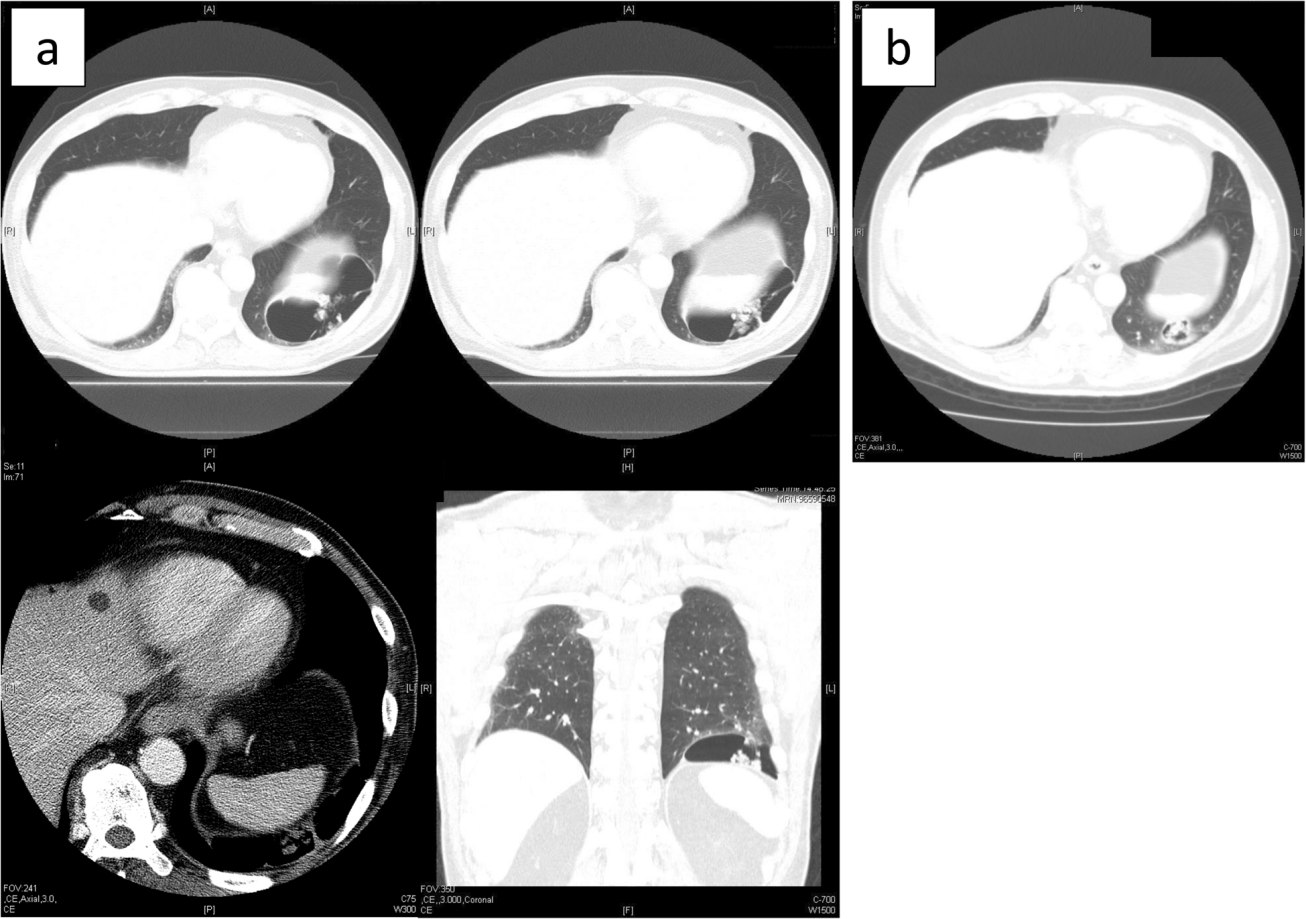
Chest CT showed a 9-cm cystic tumor in the left lower lobe, with small nodules aggregated in the cyst. The small nodules were slightly enhanced on contrast-enhanced CT (**a**). The cystic tumor had been found on abdominal CT 12 years previously (**b**)

**Fig. 3:**
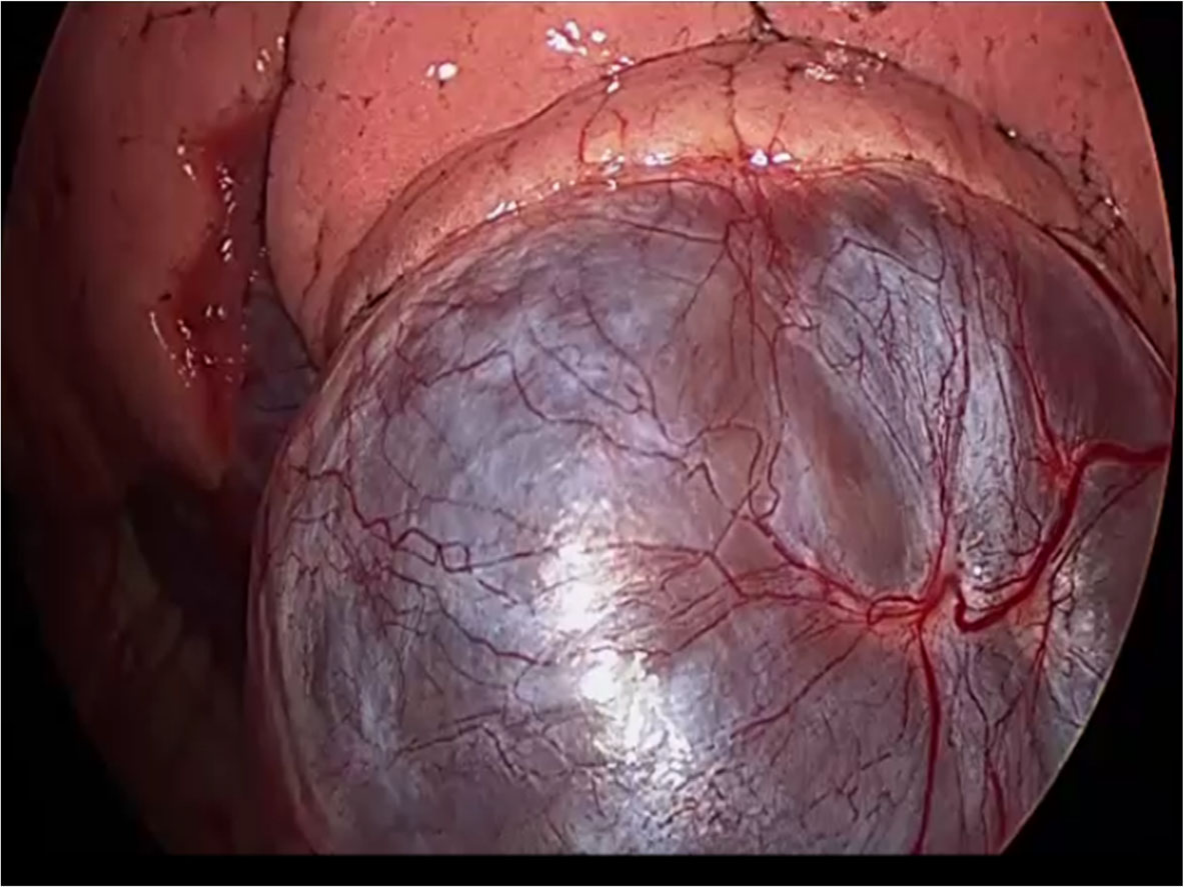
The intraoperative findings. A cystic tumor was observed in the left lower lobe; no invasion of the surrounding organs was found. The tumor stalk could not be confirmed due to the limitation of the surgical field in the thoracic cavity by the large cystic tumor itself

**Fig. 4:**
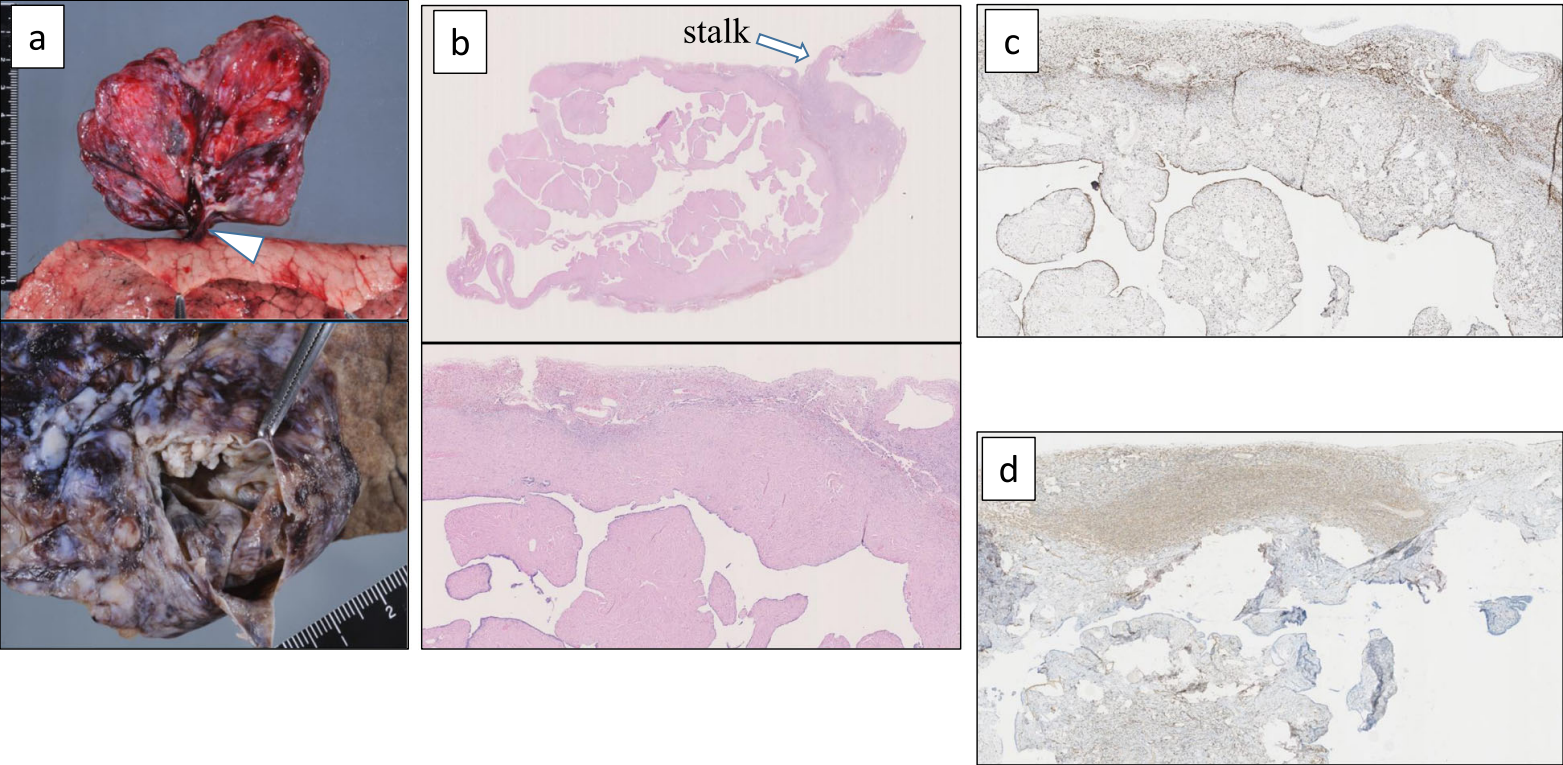
The histopathological findings of the resected cystic tumor. A pedunculated cystic tumor was connected to the pleura with a stalk (white arrow head), and white polypoidal masses were found within the cystic tumor (**a**). Uniform fibroblastic spindle cell proliferation and marked cystic degeneration were found; all cyst walls were formed of the same tumor cells (**b**). Immunohistochemical staining revealed that the tumor cells were positive for STAT6, CD34 (**c**, **d**)

The patient was a 67-year-old asymptomatic man who was referred to our hospital due to an abnormal shadow on a chest X-ray (Fig. 1). Chest CT showed a 9-cm cystic tumor in the left lower lobe, with small nodules aggregated in the cyst. The small nodules were slightly enhanced on contrast-enhanced CT (Fig. 2a). The cystic tumor was found on abdominal CT 12 years previously (Fig. 2b), when the patient’s blood beta-D-glucan level was found to be slightly elevated (56.5 pg/mL). The small nodules were judged to be a fungus ball in the giant bullae. Based on these examinations, the patient was diagnosed with pulmonary aspergillosis. Because the lesion was only localized in the cyst and not in other lobes of the lungs, it was considered that complete surgical resection of the cystic tumor was appropriate, and left basal segmentectomy was performed (Fig. 3). The tumor was a pedunculated cystic tumor, connected to the pleura with a stalk, and white polypoidal masses were found within the cystic tumor (Fig. 4a). Microscopy revealed uniform fibroblastic spindle cell proliferation and marked cystic degeneration; all cyst walls were formed of the same tumor cells (Fig. 4b). Immunohistochemical staining revealed that the tumor cells were positive for STAT6, CD34, CD99, and BCL2 (Fig. 4c, d). Based on these findings, the tumor was diagnosed as SFTP with cystic degeneration. The mitosis count was 0–1/10 in high power fields, and the tumor was judged to have a low risk of recurrence. The postoperative course was good without complications. There has been no recurrence in the 2 months after surgery.

## Discussion

Solitary fibrous tumors of the pleura (SFTPs) are relatively rare, with an incidence of < 3 per 100,000 hospital patients, and less than 1000 cases reported in the literature [4]. They account for < 5% of all pleural tumors [1]. SFTPs affect males and females equally, and occurs in all age groups, although the mean age of reported cases is 50–57 years [5].

Half of the patients (43% to 67%) are asymptomatic at the time of diagnosis. The larger the tumor, the more likely that symptoms such as cough, chest pain, or dyspnea will be present [3, 6, 7]. In our case, the patient had no symptom, despite having a 9-cm tumor; this may be because the shape of the tumor was cystic.

Chest CT is the key examination for diagnostic imaging and surgical planning. In most cases of SFTP, both benign and malignant varieties, CT demonstrates a homogeneous well-defined, non-invasive, lobular, soft-tissue mass, adjacent to the chest wall or within a fissure, showing an obtuse angle with the pleural surface [3, 6]. In contrast to many of the reports on SFTP, our case was considered extremely rare. The first review of SFTP by England et al. reported that cysts were present in 13% tumors, and more prominent at the base of the tumor near its pleural attachment [5]. The cysts described in this review were part of the tumor and did not include cases in which the entire tumor showed cystic degeneration. Furthermore, in the present case, small nodules aggregating in the cyst were seen; these were judged to be aspergilloma because the patient’s blood level of beta-D-glucan was slightly elevated.

Macroscopic observation of the present case revealed a pedunculated cystic tumor connected to the pleura with a stalk. A stalk of pleural attachment is seen approximately 82% of SFTPs [8]. Intraoperatively, the tumor stalk could not be confirmed due to the limitation of the surgical field in the thoracic cavity by the large cystic tumor itself and because we were trying to avoid rupturing the cyst. But, we should have considered the potential for unexpected tumors because the cystic tumor without any adhesion was quite unusual as pulmonary aspergillosis. Further observation of the tumor may have prevented excessive lung resection. If we had noticed the stalk during the operation, we would have considered partial lung resection.

Histologically, SFTP is characterized by a multiplicity of growth patterns, described as “pattern-less pattern.” The two major histological patterns of SFTP are (i) solid spindle and (ii) diffuse sclerosing [9]. These are admixed in varying proportions. Immunohistochemical staining is required for the diagnosis of SFTP; the tumor cells are positive for STAT6, CD34, CD99, and BCL2, whereas they are negative for desmin, S-100, and alpha-SMA. In our case, although microscopy revealed typical findings in individual tumor cells, the findings were quite atypical in that the tumor cells had cystic degeneration, and the entire cyst wall was formed by tumor cells, which differed from the development of SFTP in a pleural cyst. The mechanism of cystic degeneration of SFTP with pathology in our case is unknown. As shown in Fig. 2b, the tumor had developed at least 12 years previously and cystic degeneration was already present at the time. Based on these findings, we considered that the SFTP of our case had not formed a solid tumor and had cystic degeneration at an early stage. In addition, the cyst was filled with air, not liquid, and it was considered that the cystic tumor had connected with the peripheral airways. The check-valve mechanism may have caused cystic degeneration during the growth of the tumor. On the other hand, it has been reported that genetic alterations analyzed by next-generation sequencing could be involved in malignant transformation in SFTP [10], and genetic alterations may also be involved in cystic degeneration. To the best of our knowledge, this is the first report of SFTP with cystic degeneration. It is expected that the mechanism of cystic degeneration of SFTP will be elucidated by the accumulation of further cases and progress in gene analysis techniques.

## Conclusion

We herein reported an extremely rare case of SFTP with cystic degeneration.

## Data Availability

Not applicable.

## Abbreviations

SFTP: Solitary fibrous tumor of the pleura
CT: Computed tomography

## References

[1] Balduyck B, Lauwers P, Govert K, Hendriks J, Maeseneer MD, Paul VS (2006). Solitary fibrous tumor of the pleura with associated hypoglycemia: Doege-Potter syndrome: a case report. J Thorac Oncol..

[2] Abu AW (2012). Solitary fibrous tumours of the pleura. Eur J Cardiothorac Surg..

[3] Cardinale L, Ardissone F, Volpicelli G, Solitro F, Fava C (2010). CT signs, patterns and differential diagnosis of solitary fibrous tumors of the pleura. J Thorac Dis..

[4] Ichiki I, Kakizoe K, Hamatsu T, Matsuyama A, Suehiro T, Tanaka F (2017). Solitary fibrous tumor of the lung: a case report. Surg Case Rep..

[5] England DM, Hochholzer L, McCarthy MJ (1989). Localized benign and malignant fibrous tumors of the pleura. A clinicopathologic review of 223 cases. Am J Surg Pathol..

[6] Robinson LA (2006). Solitary fibrous tumor of the pleura. Cancer Control..

[7] Sung SH, Chang JW, Kim J, Lee KS, Han J, Park SI (2005). Solitary fibrous tumors of the pleura: surgical outcome and clinical course. Ann Thorac Surg..

[8] Inoue S, Fujino S, Kontani K, Sawai S, Tezuka N, Hanaoka J (2002). Thoracoscopic resection for solitary fibrous tumor of the pleura: report of three cases. Jpn J Chest Surg..

[9] Moran CA, Suster S, Koss MN (1992). The spectrum of histologic growth patterns in benign and malignant fibrous tumors of the pleura. Semin Diagn Pathol..

[10] Zuoqing S, Fan Y, Yingguo Z, Ping F, Guowei L, Chao L (2018). Surgical therapy and next-generation sequencing-based genetic alteration analysis of malignant solitary fibrous tumor of the pleura. Onco Targets Ther..

